# Epstein–Barr virus, Cytomegalovirus, and Herpes Simplex-1/2 reactivations in critically ill patients with COVID-19

**DOI:** 10.1186/s40635-024-00624-9

**Published:** 2024-04-22

**Authors:** Alessia Mattei, Lorenzo Schiavoni, Elisabetta Riva, Massimo Ciccozzi, Roberta Veralli, Angela Urselli, Vincenzo Citriniti, Antonio Nenna, Giuseppe Pascarella, Fabio Costa, Rita Cataldo, Felice Eugenio Agrò, Massimiliano Carassiti

**Affiliations:** 1 Unit of Anaesthesia and Intensive Care, Fondazione Policlinico Universitario Campus Bio-Medico, 00128 Rome, Italy; 2 Unit of Virology, Università Campus Bio-Medico, 00128 Rome, Italy; 3 Unit of Medical Statistics and Molecular Epidemiology, Università Campus Bio-Medico, Rome, Italy; 4 Unit of Clinical Laboratory Science, Unit of Virology, Fondazione Policlinico Universitario Campus Bio-Medico, Via Alvaro del Portillo, 200, 00128 Rome, Italy; 5 Cardiac Surgery Unit, Fondazione Policlinico Universitario Campus Bio-Medico, 00128 Rome, Italy

**Keywords:** HSV, Critically ill, Mortality, COVID-19, Pneumonia, Bacteremia

## Abstract

**Objectives:**

To assess the incidences of Herpes Simplex-1 and 2 (HSV-1, HSV-2), Cytomegalovirus (CMV), Epstein–Barr Virus (EBV) reactivations in critically ill COVID-19 patients. To determine the association between viral reactivation and in-hospital mortality, Intensive Care Unit Bloodstream infection (ICU–BSI), ventilator-associated pneumonia (VAP).

**Design:**

Observational retrospective cohort study.

**Setting:**

COVID-19 Intensive Care Unit.

**Patients:**

From November 2020 to May 2021, one hundred and twenty patients with COVID-19 severe pneumonia were enrolled and tested for HSV-1, HSV-2, CMV and EBV at the admission in ICU and weekly until discharge or death. The presence of VAP and ICU–BSI was evaluated according to clinical judgement and specific diagnostic criteria.

**Measurements and main results:**

One hundred and twenty patients were enrolled. Multiple reactivations occurred in 75/120 (63%) patients, single reactivation in 27/120 patients (23%). The most reactivated Herpesvirus was EBV, found in 78/120 (65%) patients. The multivariate analysis demonstrated that viral reactivation is a strong independent risk factor for in-hospital mortality (OR = 2.46, 95% CI 1.02–5.89), ICU–BSI (OR = 2.37, 95% CI 1.06–5.29) and VAP (OR = 2.64, 95% CI 1.20–5.82).

**Conclusions:**

Human Herpesviruses reactivations in critically ill patients with COVID-19 severe Pneumonia are associated with mortality and with a higher risk to develop both VAP and ICU–BSI.

**Supplementary Information:**

The online version contains supplementary material available at 10.1186/s40635-024-00624-9.

## Key points

**Question**: The impact of Human Herpesviruses reactivations on the morbidity and mortality of COVID-19 critically ill patients is controversial.

**Findings**: Critically ill patients with COVID-19 are at high risk for both EBV, HSV and CMV reactivations.

**Meanings**: Further large prospective studies will be necessary to clarify if the presence of active viral replication must be considered causal agent affecting mortality or the indirect manifestation of immune paralysis in the worst patients.

**Supplementary Information:**

The online version contains supplementary material available at 10.1186/s40635-024-00624-9.

## Introduction

The impact of Human Herpesviruses (HHVs) reactivations in critically ill patients remains highly debated. No clear relationship has been shown between the development of HHVs reactivations and deleterious consequences regarding morbidity (prolonged mechanical ventilation, bacterial superinfection and/or hospital stay) and mortality. Therefore, there is no definitive answer to the question whether viral reactivation is only one of the hallmarks of the severity of an underlying disease or it is directly responsible for organ disease such as pneumonia [1–4] and colitis [5].

However, by modulating immune function and anti-inflammatory response, these viruses might be responsible for the occurrence of bacterial superinfections and/or the worsening of pre-existing inflammatory processes such as acute respiratory distress syndrome (ARDS) with important therapeutic implications.

The clinical management remains highly heterogenous in terms of prophylactic and therapeutic approaches [6]. To date, no clinical recommendations have been provided regarding the need for specific HHVs reactivations treatments in critically ill patients.

Regarding critically ill patients affected by severe COVID-19 pneumonia, few studies investigated the impact of HHVs reactivations, such as Cytomegalovirus (CMV), Epstein–Barr Virus (EBV), Herpes Simplex Viruses (HSV1–2) reactivation on mortality and morbidity, with still largely elusive conclusions in terms of prognostic implications [7–10].

This study aims to analyze the impact of HHVs reactivation (CMV; EBV; HSV1–2) in patients with severe COVID-19 pneumonia primarily on mortality, and secondarily on ventilator-associated pneumonia (VAP) and intensive care unit blood stream infection (ICU–BSI).

## Patients and methods

In this monocentric retrospective cohort study, we included 120 patients admitted for COVID-19 infection between November 2020 and May 2021 in our dedicated 13-beds ICU (Intensive Care Unit COVID-19 department, “Policlinico Universitario Campus Bio-Medico di Roma", Italy). The study received approval by Ethical Committee of Fondazione Policlinico Universitario Campus Bio-Medico on June 22nd, 2022 with protocol number 46.22. Ethical approval stated that study plan is according to national and international ethical standards on medical research on human beings as established by Helsinki Declaration, Institutional Council of Harmonization/Good Clinical Practice, and Oviedo Convention.

Study and manuscript were designed according to the STROBE checklist.

Given the non-interventional nature of the study, no specific patient’s informed consents were required.

At the admission, all patients were re-tested for COVID-19 infection by nucleic acid detection (Real-Time Reverse Transcriptase Polymerase Chain Reaction, RT-PCR) in nasopharyngeal swab, in accordance with World Health Organisation (WHO) criteria [11], and positivity was confirmed in all cases. All patients were treated with corticosteroids according to WHO guidelines [11].

Patients with pre-existing immunodepression (defined as hematological disease, EBV-induced malignancy, having received immunosuppressive therapies in the last 3 months or having immunodeficiency syndromes) were not included in the study.

At the admission, blood samples and bronchoalveolar lavage were collected for PCR testing for EBV, CMV HSV-1 and HSV-2. PCR testing was repeated periodically every week.

EBV and CMV reactivation were defined as the detection of DNA levels higher than 1000 IU/ml in real-time quantitative PCR from peripheral blood according to previous publication [12].

HSV reactivation was defined as the detection of a virus load ≥ 130 copie/ml in plasma.

Patients enrolled did not received anti-cytokine antibodies that lie tocilizumab or anakinra.

Previous use of remdesivir was not considered an exclusion criterion, if administered before ICU admission and in accordance with treatment guidelines.

Primary endpoint of the study was the evaluation of in-hospital mortality and to report the incidence of CMV, EBV, HSV-1 and HSV-2 reactivation in critically ill patients with COVID-19 pneumonia.

Secondary endpoints include the occurrence of VAP and ICU–BSI. In addition, we have investigated the impact of lymphocyte count and C-reactive protein (CRP) as hypothetical predictors of reactivation.

Our cohort of patients, given their critical illness, was assumed to be at high risk of developing HHVs reactivations and where, therefore, monitored during their COVID–ICU stay. HHVs DNAemia was monitored with RT-PCR at the admission and once a week until discharge from COVID–ICU or death. CMV, EBV, HSV-1 and HSV-2 DNA were extracted from 500 μL of plasma samples by using ALTOSTAR AM16 Instrument and detection was performed by quantitative RT-PCR (AltoStar® CMV PCR Kit 1.5, AltoStar® EBV PCR Kit 1.5 and AltoStar® HSV1/2 PCR Kit 1.5, Altona Diagnostics, Germany). Lowest limit of sensitivity for the related assay was 200 UI/mL for CMV and EBV and 130 cp/mL for HSV1/2, respectively.

VAP was defined as a pneumonia occurring in mechanically ventilated patients for at least 48 h [13]. The following three criteria had to be fulfilled: (I) new or progressive and persistent infiltrates or consolidation or cavitation; (II) pathological body temperature (< 36 °C or > 38 °C) or white blood cell count < 4 or > 12 × 10^3^ cells/mm3; and (III) either the new onset/increase of purulent aspirates or worsening gas exchange [13, 14]. In addition to clinical criteria, a lower respiratory tract sample (bronchoalveolar lavage, BAL) was performed to improve the diagnostic accuracy and to focus and narrow the antibiotic therapy [13]; the diagnostic threshold for BAL was of 10^4^ CFU/ml.

ICU–BSI was defined as the occurrence of a bacteriemia at least 48 h after ICU admission. Typical skin contaminants (i.e., coagulase-negative staphylococci [CoNS]) were included only if ≥ 2 blood cultures showed the same phenotype on separate samples drawn within a 48-h period, or ≥ 1 blood culture positive for clinical sepsis, without any other infectious process, and with an antibacterial agent just initiated by the attending physician [9, 15].

Based on clinical data and physicians’ judgement, patients were screened both for bloodstream and for pulmonary infection. CRP and lymphocyte count were performed daily during COVID–ICU stay. Lymphocyte count < 300 cells/µL was fixed as marker of severe lymphopenia. Data were collected by trained physicians: medical history, epidemiological data, laboratory data and outcomes were gathered for all patients from laboratory electronic medical records and clinical digital diary. All patient’s data were anonymized and stored in a password protected personal computer.

### Statistical analysis

Categorical variables are expressed as frequencies and percentages and compared using Chi-squared test or Fisher’s exact test, as appropriate. Continuous variables were checked for normality with Kolmogorov–Smirnov test. Normally distributed variables are shown as mean and standard deviation and compared with parametric tests (Student *t* test). Not-normally distributed variables are presented as median and interquartile range. Regression analysis was performed for binary outcomes (in-hospital mortality) using logistic regression to estimate the effect. Results of the univariable analysis were adjusted for age and SAPS-II (Simplified Acute Physiology Score II) score, and the resulting multivariable model was presented. Variables with *P* value < 0.200 at univariable analysis were included in the multivariable model. Post-estimation tests (c-statistic and Hosmer–Lemeshow test) were performed to assess model fit. Considering patients and events, no more than three variables could have been included in a multivariable model to produce reliable data and avoid model overfit [15]. Statistical analysis was performed with STATA ver 17 (personal licence).

## Results

A total number of 120 patients were included in this analysis. Demographic data are shown in Additional file 1: Table S1. Median age was 61 years [range 55–71] and most of patients were of male gender (88%, *n* = 106). Median length of stay (LOS) in our COVID–ICU was 18 days [range 9.25–37.75] and median SAPS-II was 35 [range 29–42]. COVID-19 pneumonia was managed with high flow nasal oxygen (HFNO) cannula and/or non-invasive ventilation (NIV) and/or invasive mechanical ventilation; 45 patients (38%) required extracorporeal membrane oxygenation (ECMO) (Additional file 1: Table S1).

Remdesivir was administered to 66 patients (46%) before ICU admission; no patients received antiviral therapy before reactivation was diagnosed.

In-hospital events are summarized in Supplementary Data, Table 2. Multiple reactivations occurred in 75 patients (63%), while single-virus reactivation occurred in 23% of cases (27 patients). At admission none of patients tested positive for CMV, EBV, HSV-1 and HSV-2 in peripheral blood. Viruses’ detection was succeeding to COVID-19 pneumonia for more than a week and was all considered post-admission re-activations. Among them, the most reactivated HHVs was EBV (65%, *n* = 78) followed by CMV (61%, *n* = 73). HSV-1 and HSV-2 reactivation were found in 58 (48%) and 3 (2.5%) patients, respectively. The paucity of data regarding HSV-2 reactivation prevented any further statistical evaluation due to the impossibility to reach a statistical significance. Time from ICU admission to first positive DNAemia for each virus and details about specific viral reactivation and mortality in patients with reactivation are shown in Table 1. Multiple reactivations (i.e., co-reactivations) were concomitant in 27 patients (22.5%) and consecutive in 48 patients (40.0%).

**Table 1:** Specific viral reactivation and mortality

	CMV	EBV	HSV-1	HSV-2
Reactivation (%)	61%	65%	48%	2.5%
Mortality among reactivated patients (%)	67%	64%	66%	100%
Media delay between ICU admission and DNAemia detection (days [IR])	8 [3–13]	4 [2–7]	5 [3–8]	

*CMV* (Cytomegalovirus), *EBV* (Epstein–Barr Virus), *HSV-1* (Herpes Simplex Virus 1), *HSV-2* (Herpes Simplex Virus 2)

Viral reactivation was compared with clinical endpoints (Table 2): it was significantly associated with VAP (*P* = 0.012), even viral reactivation in absence of VAP was observed in 29 patients (24%).

**Table 2:** Clinical endpoints in patients with or without viral reactivation

	No reactivation *N* = 45	Viral reactivation *N* = 75	*P* value
In-hospital death	22 (49%)	52 (69%)	0.026
ICU–BSI	19 (42%)	45 (60%)	0.059
VAP	17 (38%)	46 (61%)	0.012
LOS, days	12 [6–19]	27 [13–42]	0.001

*ICU–BSI* (Intensive Care Unit Bloodstream Infections), *VAP* (Ventilator-Associated Pneumonia), *LOS* (Length of Stay)

Details of specific viral reactivation and VAP are shown in Table 3. Notably, the association between reactivation and VAP is sustained by CMV reactivation. In addition, multiple reactivations were significantly associated with VAP (46 out of 63 VAPs occurred among the 75 patients with any reactivation, *P* = 0.012).

**Table 3:** Specific viral reactivation and VAP

	VAP in patients with viral reactivation	Viral reactivation without VAP	VAP without reactivation	*P* value
CMV, 73 patients	44 (70%)	29 (40%)	19 (30%)	0.034
EBV, 78 patients	45 (71%)	33 (42%)	18 (43%)	0.122
HSV-1, 58 patients	34 (54%)	24 (41%)	29 (46%)	0.196
HSV-2, 3 patients	3 (4.8%)	0 (0.0%)	60 (95%)	0.097

*VAP* (Ventilator-Associated Pneumonia), *CMV* (Cytomegalovirus), *EBV* (Epstein–Barr Virus), *HSV-1* (Herpes Simplex Virus 1), *HSV-2* (Herpes Simplex Virus 2)

A significant trend was found in comparing lymphocyte count (< 300 U/uL, marker of immunodepression) and viral reactivation. Reactivation occurred in 26.6% among patients with lymphocyte count > 300 U/uL and 43.3% among patients with lymphocyte count < 300 U/uL, P = 0.078. CRP values were lower among patients with reactivation, with a median value of 2.8 mg/dL (0–12.1) in reactivated vs 10.8 mg/dL (4.5–19.6) in non-reactivated (*P* = 0.001).

The evaluation of ICU–BSI and specific viral reactivation showed that only EBV reactivation was significantly associated with ICU–BSI (*P* = 0.038), while other viral reactivations did not achieve statistical significance (Table 4). Despite lack of statistical significance, ICU–BSI and viral reactivation might be related: among 75 patients with reactivation, 45 (60%) had ICU–BSI and ICU–BSI was found in 19 non reactivated patients (42%) (*P* = 0.059) (Table 2).

**Table 4:** Specific viral reactivation and ICU–BSI

	ICU–BSI in patients with viral reactivation	Viral reactivation without ICU–BSI	ICU–BSI without reactivation	*P* value
CMV, 73 patients	44 (60%)	29 (40%)	20 (43%)	0.058
EBV, 78 patients	47 (60%)	31 (40%)	17 (41%)	0.038
HSV-1, 58 patients	32 (55%)	26 (45%)	32 (52%)	0.696
HSV-2, 3 patients	2 (68%)	1 (33%)	62 (53%)	0.639

ICU–BSI (Intensive Care Unit Bloodstream Infections), CMV (Cytomegalovirus), EBV (Epstein–Barr Virus), HSV-1 (Herpes Simplex Virus 1), HSV-2 (Herpes Simplex Virus 2)

Regression analysis was, therefore, performed for primary (in-hospital death) and secondary endpoints (ICU–BSI and VAP) considering overall and specific (CMV, EBV and HSV-1) reactivation. Univariate logistic regression analysis (Table 5) showed that viral reactivation was associated with in-hospital mortality (Odds Ratio, OR = 2.36, *P* = 0.027); as for ICU–BSI, a significant trend was found driven by EBV reactivation (OR 2.23, *P* = 0.040). Reactivation (any viral reactivation) carried a risk for VAP (OR = 2.61, *P* = 0.013). In addition, regression analysis confirms the association between CMV reactivation and VAP (*P* = 0.035) and the relationship between EBV reactivation and ICU–BSI (*P* = 0.040).

**Table 5:** Clinical endpoints and viral reactivation: univariate analysis

	Any viral reactivation	CMV	EBV	HSV-1
In-hospital death	2.36 (1.10–5.07) *P* = 0.027	1.79 (0.84–3.81) *P* = 0.127	1.34 (0.62–2.889) *P* = 0.455	1.37 (0.65–2.88) *P* = 0.402
ICU–BSI	2.05 (0.97–4.35) *P* = 0.060	2.05 (0.97–4.31) *P* = 0.059	2.23 (1.04–4.79) *P* = 0.040	1.15 (0.56–2.37) *P* = 0.696
VAP	2.61 (1.22–5.59) *P* = 0.013	2.23 (1.06–4.72) *P* = 0.035	1.81 (0.85–3.88) *P* = 0.122	1.61 (0.78–3.32) *P* = 0.195

Data are shown as odds ratio (95% confidence interval)

*ICU–BSI* (Intensive Care Unit Bloodstream Infections), *VAP* (Ventilator-Associated Pneumonia), *CMV* (Cytomegalovirus), *EBV* (Epstein–Barr Virus), *HSV-1* (Herpes Simplex Virus 1)

Considering literature data, results of univariable analysis were adjusted for age and SAPS II score. The multivariable model confirmed that viral reactivation is a strong independent risk factor for in-hospital death (OR 2.46, *P* = 0.045), ICU–BSI (OR 2.37, *P* = 0.034) and VAP (OR 2.64, *P* = 0.016) (Table 6). Sub-analysis for specific viral reactivation confirmed the association between CMV and VAP as well as the association between EBV and ICU–BSI.

**Table 6:** Clinical endpoints and viral reactivation: adjusted analysis

	Any viral reactivation	CMV	EBV	HSV-1
In-hospital death	2.46 (1.02–5.89) *P* = 0.045 Age: 1.04 (1.01–1.07), *P* = 0.042 SAPS: 1.34 (1.12–1.56), *P* = 0.034 AUC = 0.758 HL = 0.37	2.28 (0.96–5.44) *P* = 0.062 Age: 1.05 (1.00–1.09), *P* = 0.053 SAPS: 1.39 (1.10–1.69), *P* = 0.032	1.16 (0.48–2.79) *P* = 0.736 Age: 1.06 (0.92–1.20), *P* = 0.071 SAPS: 1.42 (0.82–2.08), *P* = 0.128	1.13 (0.49–2.60) *P* = 0.736 Age: 1.05 (0.84–1.31), *P *= 0.375 SAPS: 1.50 (0.78–2.31), *P* = 0.481
ICU–BSI	2.37 (1.06–5.29) *P* = 0.034 Age: 1.12 (1.05–1.20), *P* = 0.029 SAPS: 1.25 (1.14–1.35), *P* = 0.037 AUC = 0.646 HL = 0.37	2.38 (1.08–5.22) *P* = 0.031 Age: 1.15 (1.08–1.23), *P* = 0.028 SAPS: 1.32 (1.10–1.55), *P* = 0.039	2.41(1.08–5.37) *P* = 0.031 Age:1.07 (1.02–1.12), *P* = 0.047 SAPS: 1.44 (0.84–2.12), *P* = 0.124	1.10 (0.52–2.34) *P* = 0.786 Age: 1.83 (0.95–2.78), *P* = 0.087 SAPS: 2.18 (0.52–3.83), *P* = 0.420
VAP	2.64 (1.20–5.82) *P* = 0.016 Age: 1.42 (1.21–1.63), *P* = 0.027 SAPS: 1.74 (1.29–2.20), *P* = 0.019 AUC = 0.649 HL = 0.47	2.33 (1.08–5.02) *P* = 0.030 Age:1.51 (1.24–1.78), *P* = 0.028 SAPS: 1.68 (1.29–2.01), *P* = 0.035	1.73 (0.79–3.74) *P* = 0.164 Age: 1.37 (0.95–1.75), *P* = 0.058 SAPS: 1.89 (1.14–2.73), *P* = 0.039	1.59 (0.75–3.35) *P* = 0.220 Age: 1.72 (0.75–2.57), *P* = 0.671 SAPS: 2.58 (0.85–4.18), *P* = 0.394

Data are shown as odds ratio (95% confidence interval). Data are adjusted for age and SAPS score

*AUC* Area Under the ROC Curve. *HL* *P* value associated with Hosmer–Lemeshow test. *ICU–BSI* (Intensive Care Unit Bloodstream Infections), *VAP* (Ventilator-Associated Pneumonia)

## Discussion

In this study, a total number of 120 patients admitted to our ICU for severe COVID-19 pneumonia were screened for HHVs reactivation (CMV, EBV, HSV-1 and HSV-2).

We found a high incidence of both multiple and single reactivation; specifically, CMV, EBV, HSV-1 and HSV-2 were detected in blood samples in 61%, 65%, 48% and 2.5% of patients, respectively.

Epstein–Barr virus reactivation was the most frequent and occurred earliest among the tested Herpetic viruses. These results are consistent with previous study [8, 10].

The higher proportion of EBV reactivation is in line with previous findings in critically ill COVID-19 and non-COVID-19 patients, where the use of high-dose corticosteroid treatment has been reported as a risk factor for HHVs reactivations [10, 12].

The incidences found in our experience are higher compared to the frequencies reported in larger studies for septic-shock patients (18–40% for CMV and 10–24% for HHV-6) [1, 2, 5, 9, 12]and in other studies conducted on COVID-19 critically ill patients [8, 17].

Our patients had several risk factors for viral reactivations: the first one is COVID-19 infection which is responsible itself for a tendency to develop lymphopenia. The second one is corticosteroid therapy, administered according to WHO guidelines. The third is the critical illness itself which is an independent risk factor for HS reactivations even in absence of both COVID-19 pneumonia and immunosuppression [12, 18]. Therefore, we believe that all these risk factors could be synergistically responsible for both the high incidence of viral reactivations and for mortality.

We aimed to clarify the prognostic implication of HHVs reactivations, which is still elusive and makes its clinical management still heterogeneous [6]; we, therefore, assessed the impact of these reactivations on mortality and, as previously reported, a significant association was found. After adjustment for baseline characteristics, such as age and SAPS II score, viral reactivation carries a significant risk of in-hospital mortality in our cohort of COVID-19 critically ill patients.

In a retrospective study conducted by Saade et al. [7], they evaluated the incidence of HHVs reactivation in a cohort of 100 patients with severe COVID-19 pneumonia admitted to the ICU of an onco-haematology academic hospital. A total of 63 patients (63%) presented viral reactivation during the ICU stay (12% for HSV-1, 58% EBV and 19% CMV). However different from us, baseline patients’ characteristics were quite different (onco-haematology intensive care unit) and surprisingly, despite their critical illness and immunosuppression, no association between viral reactivation and mortality was found.

Another retrospective study conducted on 20 critically ill patients with COVID-19 pneumonia, confirmed the lack of association between herpetic reactivations and mortality; however, HSV-1 and HSV-2 were not included, and the sample size was smaller [8].

A strong association between HSV-1 infection and COVID-19 was demonstrated in different studies with no definitive conclusions regarding the impact on mortality. In a recent observational prospective study conducted on 153 critically ill COVID-19 patients, the incidence of HSV-1 reactivation was 26.1% with a day-60 mortality higher in patients with HSV-1 reactivation (57.5%) versus without (33.6%, *P* = 0.001) [9];

Several authors demonstrated an association between mortality and HHVs reactivation in critically ill patients [19–21]. Our findings confirm these results in a cohort of critically ill patients admitted to ICU for COVID-19 severe pneumonia. The pathophysiological mechanisms responsible for a higher mortality and morbidity might be the same described for critically ill non-COVID-19 patients: a direct cytopathic effect of HHVs and/or an impairment of the immune system leading to a higher risk for fungal and bacterial infections.

However, it is difficult to determine the causality between HHVs reactivations and mortality since many confounders might have been unmeasured, also considering the retrospective nature of this study.

The impact of HHVs reactivations on VAP has been already described in literature with an incidence up to 31.0% in VAP intubated ICU patients [22].

To our best knowledge only few studies investigated the association between HHVs reactivation and VAP in COVID-19 critically ill patients.

Different from us, Meyer et al. showed an association between HSV-1 reactivation and VAP which remained significant after adjustment on several factors [9].

Giacobbe et al., found no association between HSV-1 reactivation in bronchoalveolar fluid samples and mortality in mechanically ventilated COVID-19 patients. In line with these results, it is reasonable that HSV-1 could be a simple bystander and its presence might not reflect a direct pathogenicity [6]. Another possibility is that the association with mortality and HSV-1 reactivation in critically ill COVID-19 patients may be driven by blood reactivation and not by respiratory reactivation [9].

In our experience, HHVs reactivations were found to be associated with VAP; the association is significant in those patients who experienced CMV reactivation. This result confirms what found in a large study conducted by Girardis et al., on 431 COVID-19 critically ill patients; CMV reactivation was found to be associated with higher mortality and a high risk of developing bacterial superinfections, in particular VAP [23].

The rationale needs to be elucidated; it may be reasonable to hypothesize that viral reactivation could be responsible for both lung and/or respiratory tract injuries (leading to a deterioration of gas exchange) [9] and bacterial superinfection. Another plausible explanation is that severe COVID-19 pneumonia exposes patients both to bacterial superinfections and to an increased risk of viral reactivation because of the induced immunosuppression.

Further larger studies are warranted to clarify the role of this viral reactivation, but a practical clinical implication could be the possibility to identify patients at risk of developing superinfections among COVID-19 patients with HHVs reactivation.

A high incidence of bloodstream infection in COVID-19 patients has been described in literature, with a significant impact on mortality [24, 25].

The impact of HHVs reactivations on ICU–BSI remains unclear and further studies will be necessary to clarify. In our cohort, only EBV reactivation was found significantly associated with ICU–BSI (OR = 2.23, *P* = 0.04); the association between any viral reactivation and ICU–BSI did not reach a statistical significance. To the best of our knowledge, this is the first study suggesting a possible relationship between EBV reactivation and the occurrence of ICU–BSI. It is plausible that this finding may be related to a play of the chance due to our reduced sample size. Nevertheless, we believe that those results highlight the importance of proper surveillance and further studies are needed to address this finding.

Regarding lymphocyte count, viral reactivation occurred in 43% patients with lymphocyte count < 300 U/uL versus 27% of patient with lymphocyte count > 300 (*P* = 0.078). Despite the lack of statistical significance, this result underlines the importance of monitoring lymphocyte count during ICU stay since lymphopenia is common in critically ill patients for a wide range of risk factors [26, 27].

As previously reported, CRP values were lower among reactivated patients. Not by chance, CRP values do not reliably predict reactivation and no correlations between viremia and markers of inflammation were identified, in line with similar studies published [10].

### Limitations

This study has several limitations. First, the nature of the study; this was a single-center, retrospective cohort study. Second, the small sample size is a huge limit to reach a statistical significance; further studies will be necessary to confirm our findings. Third, results of logistic regression analysis should be interpreted with cautions considering the presence of competing risks and their interplay. We acknowledge the importance of the duration of mechanical ventilation and of the details of ventilation (oro-tracheal intubation, non-invasive ventilation, high flow nasal cannula [HFNC]). However, ventilation mode was a time-dependent variable (i.e., a patient might have received HFNC before non-invasive ventilation) and the time-to-event was not recorded; therefore, this variable was deemed unreliable for analysis, potentially leading to bias (e.g. model overfit in regression analysis, considering the available patients and the events). This might require tailored investigation with ad hoc analysis and a larger dataset. Finally, although HHVs reactivations were found to be associated with VAP, we did not investigate the impact of these viruses on the duration of mechanical ventilation.

## Conclusions

Our experience confirms the association between viral reactivation and mortality even if in our cohort most patients suffered of multiple viral reactivations.

We can, therefore, conclude that the association between mortality and viral reactivations remains unclear and further studies will be necessary to clarify if the presence of active viral replication must be considered a real risk factor or only a simple bystander [28].

### Supplementary Information

**Additional file 1: Table S1.** Demographic data and baseline characteristics. **Table S2.** In-hospital events and incidence of viral reactivation.

## Data Availability

All data are stored anonymously in local server and on paper and can be available on reasonable request.

## References

[1] Papazian L, Fraisse A, Garbe L (1996). Cytomegalovirus. An unexpected cause of ventilator-associated pneumonia. Anesthesiology.

[2] Papazian L, Doddoli C, Chetaille B (2007). A contributive result of open-lung biopsy improves survival in acute respiratory distress syndrome patients. Crit Care Med.

[3] Walton AH, Muenzer JT, Rasche D (2014). Reactivation of multiple viruses in patients with sepsis. PLoS ONE.

[4] Huang L, Zhang X, Pang L (2023). Viral reactivation in the lungs of patients with severe pneumonia is associated with increased mortality, a multicenter, retrospective study. J Med Virol.

[5] Siciliano RF, Castelli JB, Randi BA, Vieira RD, Strabelli TMV (2014). Cytomegalovirus colitis in immunocompetent critically ill patients. Int J Infect Dis IJID Off Publ Int Soc Infect Dis.

[6] Giacobbe DR, Di Bella S, Dettori S (2022). Reactivation of herpes simplex virus type 1 (HSV-1) detected on bronchoalveolar lavage fluid (BALF) samples in critically Ill COVID-19 patients undergoing invasive mechanical ventilation: preliminary results from two Italian centers. Microorganisms.

[7] Saade A, Moratelli G, Azoulay E, Darmon M (2021). Herpesvirus reactivation during severe COVID-19 and high rate of immune defect. Infect Dis Now.

[8] Simonnet A, Engelmann I, Moreau AS (2021). High incidence of Epstein-Barr virus, cytomegalovirus, and human-herpes virus-6 reactivations in critically ill patients with COVID-19. Infect Dis Now.

[9] Meyer A, Buetti N, Houhou-Fidouh N (2021). HSV-1 reactivation is associated with an increased risk of mortality and pneumonia in critically ill COVID-19 patients. Crit Care Lond Engl.

[10] Naendrup JH, Garcia Borrega J, Eichenauer DA, Shimabukuro-Vornhagen A, Kochanek M, Böll B (2022). Reactivation of EBV and CMV in Severe COVID-19-Epiphenomena or Trigger of Hyperinflammation in Need of Treatment? A large case series of critically ill patients. J Intensive Care Med.

[11] Clinical Management of COVID-19: Living Guideline. World Health Organization; 2022. http://www.ncbi.nlm.nih.gov/books/NBK582435/. Accessed 13 May 2023 35917394

[12] Ong DSY, Bonten MJM, Spitoni C (2017). Epidemiology of multiple herpes viremia in previously immunocompetent patients with septic shock. Clin Infect Dis Off Publ Infect Dis Soc Am.

[13] Torres A, Niederman MS, Chastre J (2017). International ERS/ESICM/ESCMID/ALAT guidelines for the management of hospital-acquired pneumonia and ventilator-associated pneumonia: Guidelines for the management of hospital-acquired pneumonia (HAP)/ventilator-associated pneumonia (VAP) of the European Respiratory Society (ERS), European Society of Intensive Care Medicine (ESICM), European Society of Clinical Microbiology and Infectious Diseases (ESCMID) and Asociación Latinoamericana del Tórax (ALAT). Eur Respir J.

[14] Timsit JF, Esaied W, Neuville M, Bouadma L, Mourvllier B (2017). Update on ventilator-associated pneumonia. Research.

[15] Buetti N, Ruckly S, de Montmollin E (2021). COVID-19 increased the risk of ICU-acquired bloodstream infections: a case-cohort study from the multicentric OUTCOMEREA network. Intensive Care Med.

[16] Blackstone EH (2016). Sufficient data. J Thorac Cardiovasc Surg.

[17] Le Balc’h P, Pinceaux K, Pronier C, Seguin P, Tadié JM, Reizine F (2020). Herpes simplex virus and cytomegalovirus reactivations among severe COVID-19 patients. Crit Care Lond Engl.

[18] Libert N, Bigaillon C, Chargari C (2015). Epstein-Barr virus reactivation in critically ill immunocompetent patients. Biomed J.

[19] Tuxen DV, Cade JF, McDonald MI, Buchanan MR, Clark RJ, Pain MC (1982). Herpes simplex virus from the lower respiratory tract in adult respiratory distress syndrome. Am Rev Respir Dis.

[20] Bouza E, Giannella M, Torres MV (2011). Herpes simplex virus: a marker of severity in bacterial ventilator-associated pneumonia. J Crit Care.

[21] Brenner T, Rosenhagen C, Hornig I (2012). Viral infections in septic shock (VISS-trial)-crosslinks between inflammation and immunosuppression. J Surg Res.

[22] Daubin C, Vincent S, Vabret A (2005). Nosocomial viral ventilator-associated pneumonia in the intensive care unit: a prospective cohort study. Intensive Care Med.

[23] Gatto I, Biagioni E, Coloretti I (2022). Cytomegalovirus blood reactivation in COVID-19 critically ill patients: risk factors and impact on mortality. Intensive Care Med.

[24] Giacobbe DR, Battaglini D, Ball L (2020). Bloodstream infections in critically ill patients with COVID-19. Eur J Clin Invest.

[25] Buetti N, Tabah A, Loiodice A (2022). Different epidemiology of bloodstream infections in COVID-19 compared to non-COVID-19 critically ill patients: a descriptive analysis of the Eurobact II study. Crit Care Lond Engl.

[26] Rubio I, Osuchowski MF, Shankar-Hari M (2019). Current gaps in sepsis immunology: new opportunities for translational research. Lancet Infect Dis.

[27] Clari MA, Aguilar G, Benet I (2013). Evaluation of cytomegalovirus (CMV)-specific T-cell immunity for the assessment of the risk of active CMV infection in non-immunosuppressed surgical and trauma intensive care unit patients. J Med Virol.

[28] Simoons-Smit AM, Kraan EM, Beishuizen A, Strack van Schijndel RJ, Vandenbroucke-Grauls CM (2006). Herpes simplex virus type 1 and respiratory disease in critically-ill patients: Real pathogen or innocent bystander?. Clin Microbiol Infect Off Publ Eur Soc Clin Microbiol Infect Dis.

