# Supplementary material for: Epstein–Barr virus, Cytomegalovirus, and Herpes Simplex-1/2 reactivations in critically ill patients with COVID-19
Source: Intensive Care Med Exp. 2024 Apr 22;12:40. doi: 10.1186/s40635-024-00624-9 (PMC11035506; doi:10.1186/s40635-024-00624-9)
Supplement: Supplementary file 1 — Additional file 1: Table S1. Demographic data and baseline characteristics. Table S2. In-hospital events and incidence of viral reactivation. [file 40635_2024_624_MOESM1_ESM.docx]

**Table S1. Demographic data and baseline characteristics.**

| **Demographics data** | **Patients N=120** |
| --- | --- |
| **Age, years** | 61 (55-71) |
| **LOS, days** | 18 (9.25-37.75) |
| **SAPS II** | 35 (29-42) |
| **Days on mechanical ventilations** | 14 (6-29) |
| **BMI (kg/m^2^)** | 28.1 (25.5-33.4) |
| **Male** | 106 (88%) |
| **Female** | 14 (12%) |
| **IT** | 91 (76%) |
| **NIV** | 78 (65%) |
| **HFNO** | 54 (45%) |
| **ECMO** | 45 (38%) |
| **ICU-BSI** | 64 (53%) |
| **VAP** | 63 (53%) |
| **Diabetes** | 21 (18%) |
| **COPD** | 5 (4.1%) |
| **Dyslipidaemia** | 18 (15%) |
| **Hypertension** | 55 (46%) |
| **CAD** | 18 (15%) |

LOS (length of stay), SAPS II (Simplified Acute Physiologic Score II), BMI (Body Mass Index), IT (Tracheal Intubation), NIV (Non-Invasive Ventilation), HFNO (High Flow Nasal Oxygenation), ECMO (Extracorporeal Membrane Oxygenation), ICU-BSI (Intensive Care Unit Bloodstream Infections), VAP (Ventilator Associated Pneumonia), COPD (Chronic Obstructive Pulmonary Disease, CAD (coronary artery disease).

**Table S2. In-hospital events and incidence of viral reactivation.**

| **Events** | **Patients**  **N=120** |
| --- | --- |
| **In-hospital death** | 74 (62%) |
| **ICU-BSI** | 64 (53%) |
| **HAP/VAP** | 63 (52%) |
| **Multiple reactivation** | 75 (63%) |
| **CMV** | 73 (61%) |
| **EBV** | 78 (65%) |
| **HSV-1** | 58 (48%) |
| **HSV-2** | 3 (2.5%) |
| **LOS, days** | 18 (9.5-37.5) |

ICU-BSI (Intensive Care Unit Bloodstream Infections), HAP/VAP (Hospital Acquired Pneumonia/Ventilator Associated Pneumonia), CMV (Cytomegalovirus), EBV (Epstein-Barr Virus), HSV-1 (Herpes Simplex Virus 1), HSV-2 (Herpes Simplex Virus 2), LOS (Length of Stay).
